# The Emergency Surgery Frailty Index (EmSFI): development and internal validation of a novel simple bedside risk score for elderly patients undergoing emergency surgery

**DOI:** 10.1007/s40520-020-01735-5

**Published:** 2020-11-18

**Authors:** Gianluca Costa, Laura Bersigotti, Giulia Massa, Luca Lepre, Pietro Fransvea, Alessio Lucarini, Paolo Mercantini, Genoveffa Balducci, Gabriele Sganga, Antonio Crucitti, ERASO (Elderly Risk Assessment, Surgical Outcome) Collaborative Study Group, F. Agresta, G. Alemanno, G. Anania, M. Antropoli, G. Argenio, J. Atzeni, N. Avenia, A. Azzinnaro, G. Baldazzi, G. Balducci, G. Barbera, G. Bellanova, C. Bergamini, L. Bersigotti, P. P. Bianchi, C. Bombardini, G. Borzellino, S. Bozzo, G. Brachini, G. M. Buonanno, T. Canini, S. Cardella, G. Carrara, D. Cassini, M. Castriconi, G. Ceccarelli, D. Celi, M. Ceresoli, M. Chiarugi, N. Cillara, F. Cimino, L. Cobuccio, G. Cocorullo, E. Colangelo, G. Costa, A. Crucitti, P Dalla Caneva, M. De Luca, A. de Manzoni Garberini, C. De Nisco, M. De Prizio, A. De Sol, A. Dibella, T. Falcioni, N. Falco, C. Farina, E. Finotti, T. Fontana, G. Francioni, P. Fransvea, B. Frezza, G. Garbarino, G. Garulli, M. Genna, S. Giannessi, A. Gioffrè, A. Giordano, D. Gozzo, S. Grimaldi, G. Gulotta, V. Iacopini, T. Iarussi, G. Laracca, E. Laterza, A. Leonardi, L. Lepre, L. Lorenzon, G. Luridiana, A. Malagnino, G. Mar, P. Marini, R. Marzaioli, G. Massa, V. Mecarelli, P. Mercantini, A. Mingoli, G. Nigri, S. Occhionorelli, N. Paderno, G. M. Palini, D. Paradies, M. Paroli, F. Perrone, N. Petrucciani, L. Petruzzelli, A. Pezzolla, D. Piazza, V. Piazza, M. Piccoli, A. Pisanu, M. Podda, G. Poillucci, R. Porfidia, G. Rossi, P. Ruscelli, A. Spagnoli, R. Sulis, D. Tartaglia, C. Tranà, A. Travaglino, P. Tomaiuolo, A. Valeri, G. Vasquez, M. Zago, E. Zanoni

**Affiliations:** 1 Department of Medical-Surgical Science and Translational Medicine, Sant’Andrea Teaching Hospital, “Sapienza” University of Rome, Via di Grottarossa 1035, Rome, Italy; 2 General Surgery Unit, Santo Spirito in Sassia Hospital, ASL Roma 1, Rome, Italy; 3 Division of Emergency and Trauma Surgery - Fondazione Policlinico “A. Gemelli” IRCCS, Catholic University of Sacred Heart, Rome, Italy; 4 Division of General Surgery, Cristo Re Hospital, Rome, Italy; 5 Emergency Surgery Unit, Sant’Andrea Teaching Hospital, “Sapienza” University of Rome, Via di Grottarossa 1035, Rome, Italy

**Keywords:** Frailty, Emergency surgery, Predictive tool, Procedure-specific morbidity

## Abstract

**Background:**

Frailty assessment has acquired an increasing importance in recent years and it has been demonstrated that this vulnerable profile predisposes elderly patients to a worse outcome after surgery. Therefore, it becomes paramount to perform an accurate stratification of surgical risk in elderly undergoing emergency surgery.

**Study design:**

1024 patients older than 65 years who required urgent surgical procedures were prospectively recruited from 38 Italian centers participating to the multicentric FRAILESEL (Frailty and Emergency Surgery in the Elderly) study, between December 2016 and May 2017. A univariate analysis was carried out, with the purpose of developing a frailty index in emergency surgery called “EmSFI”. Receiver operating characteristic curve analysis was then performed to test the accuracy of our predictive score.

**Results:**

784 elderly patients were consecutively enrolled, constituting the development set and results were validated considering further 240 consecutive patients undergoing colorectal surgical procedures. A logistic regression analysis was performed identifying different EmSFI risk classes. The model exhibited good accuracy as regard to mortality for both the development set (AUC = 0.731 [95% CI 0.654–0.772]; HL test *χ*^2^ = 6.780; *p* = 0.238) and the validation set (AUC = 0.762 [95% CI 0.682–0.842]; HL test *χ*^2^ = 7.238; *p* = 0.299). As concern morbidity, our model showed a moderate accuracy in the development group, whereas a poor discrimination ability was observed in the validation cohort.

**Conclusions:**

The validated EmSFI represents a reliable and time-sparing tool, despite its discriminative value decreased regarding complications. Thus, further studies are needed to investigate specifically surgical settings, validating the EmSFI prognostic role in assessing the procedure-related morbidity risk.

## Introduction

According to the United Nation Prospects Globally, by 2050 one in six people (16%) in the world will be older than 65 years [1]. Given the global increase in life expectancy and the subsequent rise in prevalence of elderly population, many efforts are focusing on the analysis of frailty state, assessing its role in predicting older patients’ outcomes. Thus, investigating this increasingly important issue, we can define frailty as a multifactorial clinical condition, not necessarily aging associated, that is characterized by a physiological reserves’ depletion in addition to a higher vulnerability to daily stressors [2].

This phenotype depends on a multi-systemic impairment with metabolic dysregulation, imbalance between various inflammatory mediators leading to an up-regulated chronic inflammatory state, as well as immunodeficiency and altered hormonal status [3].

Different operational definitions were proposed in the last two decades, attempting to identify frailty in older patients, such as the Fried’s frailty phenotype or the Rockwood and Mitnitski's Frailty Index. Although these measurements are highly valid and reliable in clinical setting, no international standardization has yet been reached and the variability of frailty measurements reflects the heterogeneity of elderly population [4].

Recent literature has demonstrated the role of preoperative frailty screening in predicting length of stay (LOS), operative risk, and surgical outcomes in elderly patients [5–7]. Never as in emergency setting is paramount to implement the decision-making process and to perform an accurate risk stratification, addressing patients’ priority.

On this path, we perform a study with the aim to develop and validate a new scoring system for the estimation of risk in older adults that underwent emergency surgery.

## Methods

### Study settings and protocol

This report originated from the FRAILESEL (Frailty and Emergency Surgery in the Elderly) study (ClinicalTrials.gov identifier: NCT02825082). The FRAILESEL is a large, nationwide, multicenter, prospective study that investigated the perioperative outcomes of patients aged ≥ 65 years who underwent emergency abdominal surgery between January 2017 and June 2018. Data regarding elderly patients discharged from the participating centers were prospectively collected. Clinical decisions, including operative technique, were based on the criteria of individual centers and attending surgeons. The investigators were informed about the objectives of the study and asked for complete details about the surgical management of acute abdomen in elderly patients following standard methods and collection protocols [8].

The final FRAILESEL Study protocol was approved by the Ethics Committees of Sapienza University and of all the centers, and by the boards of the societies involved. All parts of the study and the present manuscript have been checked and presented according to the checklist for Strengthening the Reporting of Observational Studies in Epidemiology (STROBE) [9].

#### Exclusion criteria and collected data confirmation

**Fig. 1:**
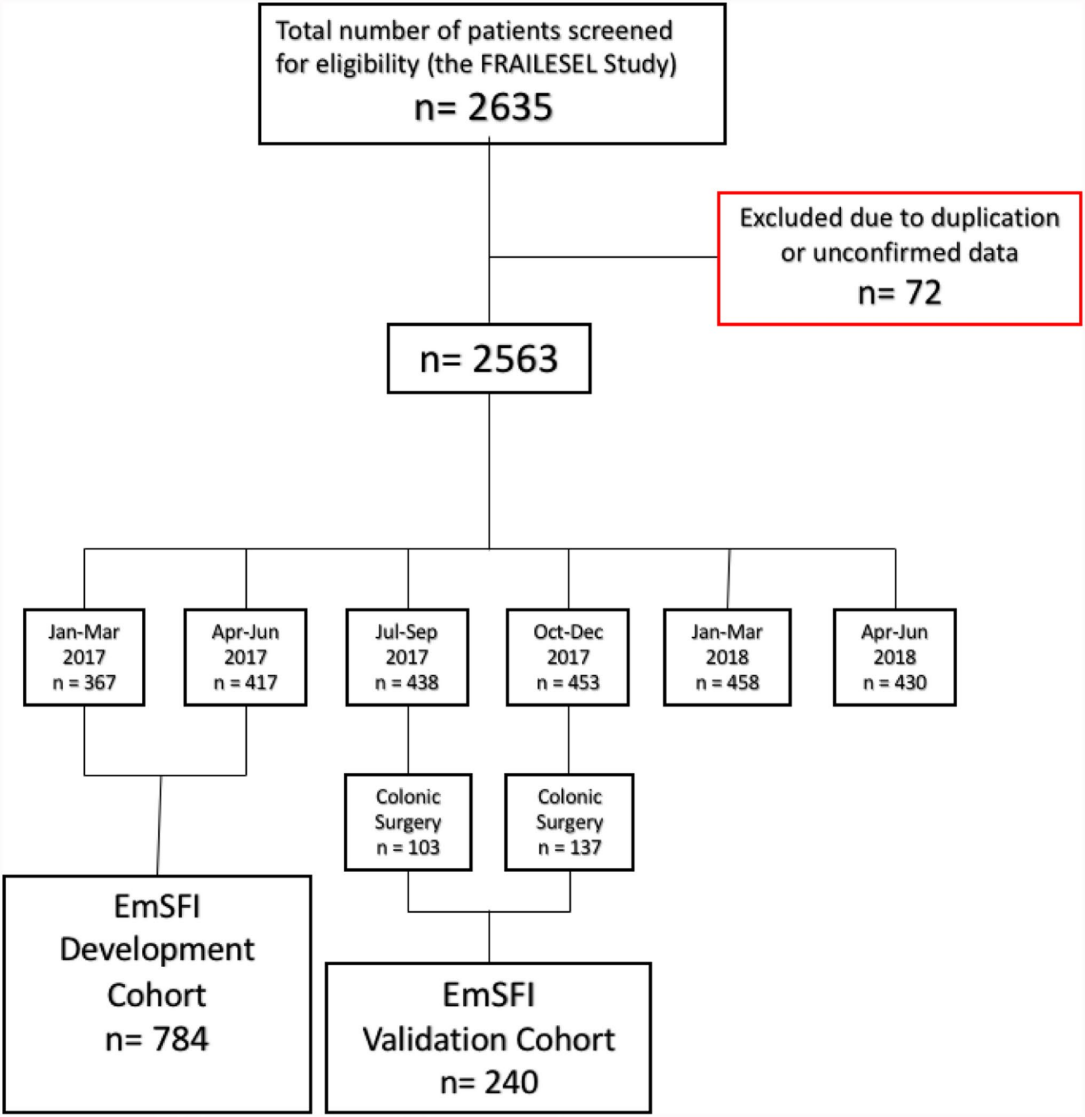
The study flow-chart according to STROBE statement

Exclusion criteria were the following: patients younger than 65 years at the day of surgery; diagnostic laparoscopy/laparotomy with no further surgical procedures performed except in case of intestinal ischemia; lack of informed consent for the study participation; emergency reoperations after elective surgery; patients already hospitalized and scheduled for the same procedure; patients participating in another trial. Submissions made by unconfirmed participants, duplicate submissions, record with more than 5% of missing data, and data submitted by residents from dual or more residency programs were excluded. Although demographic information was collected on the patients, all data were anonymized before analysis even with regard to center identification. The FRAILESEL study encompassed the final enrollment of 2635 patients, of whom 2563 were with confirmed data (Fig. 1).

### Study population and data collection

The FRAILESEL study investigates over 130 variables [8], exploring 5 domains such as patient demographic and clinical data, preoperative risk factors and operative variables, frailty condition, and postoperative outcome and follow-up. Data collected included patient demographic characteristics (age, gender, weight, height, social status), medical and surgical history (comorbidities), common preoperative biochemical blood examination (including PCR, PCT and arterial blood gas analysis), pathological features, and operative details. Comorbidity was recorded if the condition was being medically treated at the time of admission, or if previous treatment for the condition was described in the admission report. Several conditions were considered similar to the Charlson Comorbidities Index (CCI). Hypertension has been recorded but often excluded in the data analysis due to its high prevalence among the study sample, with no clinical significance according to its relative risk. Operative procedure performed and surgical diagnoses were classified according to ICD-9-CM system. The type of surgical approach takes into account open or laparoscopic procedures, including assisted or converted to open surgeries. The TNM 8th edition of UICC classification system was adopted for staging malignant tumors and preoperative risk was assessed with anesthesiologist-assigned American Society of Anesthesiologists (ASA) class.

Initially, the protocol included the assessment of frailty at arrival by emergency nurse or resident as described by Fried and coworkers in accordance to the guidelines developed by the task force of the International Conference of Frailty and Sarcopenia Research (ICFSR) [10]. However, early in the study period several difficulties emerged and only few Fried’s frailty criteria were later assessed as described below.

Postoperative complications have been reported and categorized according to the Clavien–Dindo classification system [11] by the study leader in each of the participating centers. This morbidity and mortality have been considered regardless of the time elapsed from the surgical procedure if reasonably related to it.

### The Emergency Surgery Frailty Index (EmSFI)

#### Development cohort and methodology

Seven hundred eighty-four consecutive patients enrolled between January 2017 and June 2017 represent the development cohort hereafter also referred to as Dv set. The organ or body-district affected by surgical pathologies requiring emergency procedure is listed in Table 1. On the basis of our previous studies [12, 13] and according to literature evidences, a preliminary univariate analysis was performed assessing the relationship between several items and the risk of developing postoperative complications. Any variable with *p* value ≤ 0.20 at univariate analysis was entered into a multivariate model to assess the weight of the variables consistently associated with the outcome. As already reported in the literature with regard to various emergency conditions, we confirmed the significant role of some of the items listed in the Systemic Inflammatory Response Syndrome (SIRS) definition criteria in affecting the outcome of the elderly patients undergoing emergency surgery.

**Table 1:** Organ/body district as site of surgical intervention in Dv set

Organ/body district	No. of cases (784)	%
Biliary tract	177	22.6%
Abdominal wall	129	16.4%
Large bowel	128	16.3%
Midgut miscellany	85	10.7%
Small bowel (adhesive obstruction)	83	10.6%
Upper GI	76	9.7%
Appendix	37	4.7%
Other various	69	8.8%

Then the resulting EmSFI is based on nine variables (Table 2). Assuming that the index could be suitable for both elective and emergency settings, we included the timing of surgical procedures (urgent or scheduled) as dichotomous risk variable, assigning a score of 1 for the emergency one. The other variables measured in a dichotomous manner were selected as follows: age over than 80 years, SIRS inflammatory state, and a diagnosis of solid malignancy within the last 5 years, assigning a score of 0 or 1 depending on the absence or presence of the mentioned conditions. Moreover, the remaining variables were graded assigning a score from 0 to 2 according to severity of the disease and or impairment. Among patients affected with chronic cardiovascular disease, a score of 1 is assigned in case of a positive history of cardiac disease or previous PCI (percutaneous coronary intervention) or cardiac surgery, while a score of 2 is attributed in case of myocardial infarction occurred within 6 months prior to hospital admission or in case of an acute episode of heart failure within 30 days before the hospitalization. As regards respiratory diseases a score of 1 is assigned to patients affected with mild to moderate COPD (Chronic Obstructive Pulmonary Disease), while two points are scored if a severe respiratory failure is present. Variable “*Other comorbidities*” has been rated with a modification of the Charlson Index (mCCI) where the items regarding age, solid tumor, cardiac and pulmonary diseases have been excluded. As a consequence, a score of 0 is assigned to mCCI 1–2, a score of 1 to such index between 3 and 5, and a score of 2 to the mCCI ≥ 6.

**Table 2:** Variables for calculating Emergency Surgery Frailty Index

Emergency Surgery Frailty Index (EmSFI)			
Variable	Absent		Present
Age ≥ 80 years	0		1
Emergency	0		1
SIRS	0		1
Malignancy	0		1
	Absent	Mild	Severe
Chronic cardiopathy	0	1	2
Chronic pneumopathy	0	1	2
Other comorbidities	0	1	2
Altered autonomy	0	1	2
Altered mobility	0	1	2
Maximum score = 14 points			

The assessment of Activities of Daily Living (ADL) was carried out using a reduced form of the Italian version of Barthel index [14] by which only feeding, bathing, grooming and dressing were investigated. Deepening into the whole patient’s functional autonomy area, the above criteria were combined with the mental status to simplify the EmSFI index at its best. No tests were administered to evaluate mental status thus cognitive impairment was considered as positive only if previous recognized and reported by relatives and/or caregivers. As a consequence, a score of 1 is conferred to elderly patients with alteration in the performance of daily living activities or cognitive impairment alone, whereas a score of 2 should be assigned to those patients with limitations in daily activities also with cognitive impairment.

According to Fried Frailty criteria [15], only the followings items have been considered and categorized as “Altered mobility”: slowness (slow walking speed), shrinking (unintentional weight loss) and exhaustion (self-reported). Whenever feasible, gait abnormalities were measured by walking speed (cut-off value for the time required by the patient to walk a distance of 4.5 m: 6/7 s), with or without the use of walking aids. A score of 1 is attributed to patients showing one of the above criteria, while a score of 2 is assigned in presence of more than one of such item or in case of one indicator associated with any grade of altered autonomy, when wheelchair is needed or in case of bedridden patients. If the patient was unable to walk due to his symptoms, an estimation of his gait speed was assumed based on anamnestic data and his daily activities.

To simplify the above statements, it can consider that the item “Altered autonomy” should be referred to the assessment of activities of daily living, while the item “Altered motility” has been used to outline the Fried’s frailty criteria.

Thus, the EmSFI is easily generated by summing the scores assigned for each variable with a potential maximum value of 14 points.

### Validation cohort

Two hundred forty consecutive patients undergoing emergency colonic surgery between July 2017 and December 2017 represented the internal validation cohort hereafter also referred to as Vd set. The selection criteria at the base of our decision have been driven by the fact that such cohort of patients was consistent as number with an optimal balance between benign and malignant conditions, and by the knowledge that colorectal surgery is burdened by a well-known reported outcomes in the literature after elective and emergency procedures, even in the very elderly patients [16–19].

### Statistical analysis

All statistical analyses were carried out using IBM SPSS® Statistic version 21. Scatterplot and ROC curve graphs were plotted using MedCalc version 14.8.1, MedCalc Software Ltd. Binary variables were coded as frequencies and continuous data were presented as mean ± SD (Standard Deviation), whereas other information were recorded as merely descriptive data. We used the Mann–Whitney *U* test to estimate the difference between nonparametric continuous variables. To compare frequency counts between the subdivided groups Fisher’s exact test or Chi-square test for independence were used, both including or not Yates’ continuity correction. Spearman’s rho correlation coefficient was measured to test the presence of a linear correlation between variables. A logistic regression analysis was performed to identify different morbidity and mortality risk classes according to EmSFI score. The model was evaluated for discrimination using the c-statistic and calibration using the Hosmer–Lemeshow goodness-of-fit test. In the latter, a *p* value > 0.05 reflected good agreement. Receiver operating characteristic (ROC) curve analysis was performed to test the specificity and sensitivity of the score in predicting short-term adverse post-operative outcomes, and in the determining the optimal cut-off value by the Youden’s Index (J). The c-statistic evaluates model discrimination and represents the area under the receiver operator characteristic curve. A value of 0.5 indicates that the model is equivalent to chance; a value of 1.0 indicates perfect discrimination. Statistical significance was considered with *p* values of less than 0.05.

## Results

### Study populations

Demographics were similar for both groups with a mean age of 77.82 (± 7.77) years for Dv set, within which 55.7% were men, while in Vd set 47.9% were male patients and the mean age was 77.60 (± 7.73) years. Clinical and laboratory findings of SIRS resulted comparable between the two cohorts (18.2% in Dv set and 18.8% in Vd set). Furthermore, the two sets showed similar characteristics also in terms of BMI (body mass index) and comorbidities; the latter were present in 31.25% of cases in the Dv set (245/784), as well as in 34.17% (82/784) of patients in the Vd one.

The overall morbidity (Clavien–Dindo I–IV) rate was 33.4% (262 pts) in Dv set and 42.1% (101 pts) in Vd set (*p* < 0.02). Excluding minor complications graded as I, the 30-days morbidity (Clavien–Dindo II-IV) rate was 24.2% (190 pts) in Dv set and 32.1% (77 pts) in Vd set (*p* < 0.02). Severe complications (Clavien–Dindo ≥ III) occurred in 27.8% of patients in Dv set and in 33.5% of patients in Vd set. The difference did not reach a statistically significance.

The analysis of 30-days mortality rates resulted in a non-significant difference between Dv set and Vd set reporting an overall mortality rate of 10.2% and 12.1%, respectively.

### The Emergency Surgery Frailty Index (EmSFI)

**Fig. 2:**
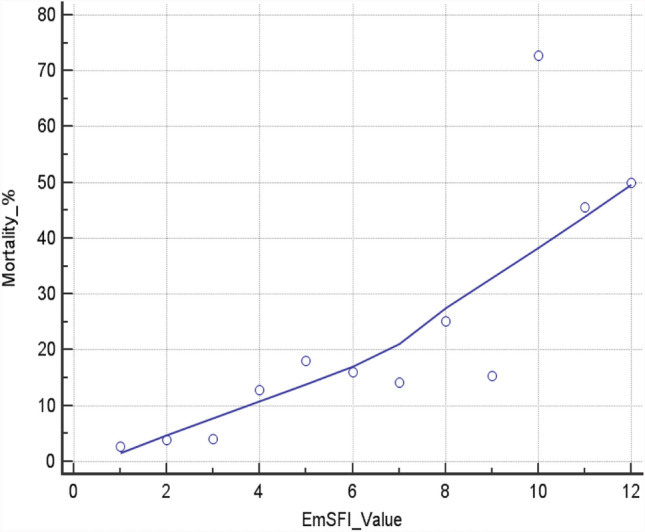
Linear correlation between EmSFI value and Mortality rate in Dv set

EmSFI mean value was 3.81 ± 2.29 in the Dv set and 4.15 ± 2.32 in the Vd set. ROC curve analysis revealed a J-index > 3 for Dv set morbidity and mortality and for Vd set mortality, while the J-index was > 2 for Vd morbidity. Correlation analysis showed that EmSFI were strongly correlated with mortality in the development set (Spearman’s rho coefficient = 0.895 [95% CI 0.661–0.970]; *p* < 0.001) (Fig. 2).

### Development cohort results

**Fig. 3:**
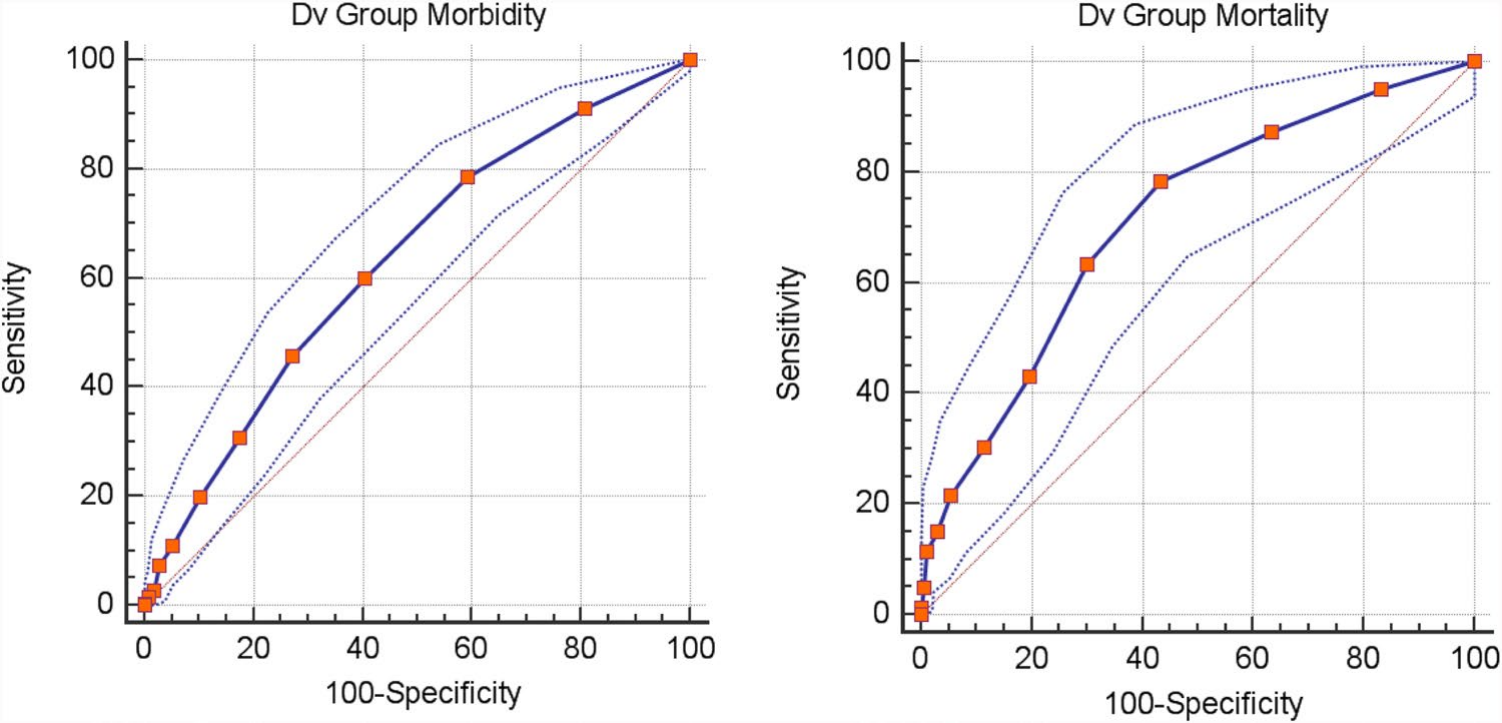
EmSFI ROC Curve of Morbidity (left) and Mortality (right) in Dv set

After logistic regression analysis, the model exhibited good discrimination ability (AUC = 0.731 [95% CI 0,654–0,772]) and good calibration (Hosmer–Lemeshow chi-square = 6,780; *p* = 0.238), reflecting a good agreement between prediction by the final model and actual observation as regard to mortality (Fig. 3; Tab. 3). As concern morbidity, the model showed a moderate discrimination ability (AUC = 0.633 [95% CI 0.593–0.673]) and good calibration (Hosmer–Lemeshow chi-square = 4,176; *p* = 0.524), reflecting a moderate agreement between prediction and actual observation (Fig. 3; Tab. 4).

**Table 3:** Hosmer–Lemeshow contingency table for mortality in Dv set

	Mortality = 0		Mortality = 1		Total
	Observed	Expected	Observed	Expected	
1	119	118,417	4	4,583	123
2	139	137,739	6	7,261	145
3	141	138,085	7	9,915	148
4	93	96,556	13	9,444	106
5	73	78,538	16	10,462	89
6	58	57,557	10	10,443	68
7	81	77,109	24	27,891	105

**Table 4:** Hosmer–Lemeshow contingency table for morbidity in Dv set

	Morbidity = 0		Morbidity = 1		Total
	Observed	Expected	Observed	Expected	
1	100	95,769	23	27,231	123
2	112	107,825	33	37,175	145
3	99	104,369	49	43,631	148
4	69	70,343	37	35,657	106
5	50	55,121	39	33,879	89
6	39	38,962	29	29,038	68
7	53	49,611	52	55,389	105

The statistical analysis allowed to stratify patients in three risk classes according to the developed index: EmSFI 1–3: low-risk class; EmSFI 4–7: moderate-risk class; EmSFI: 8–14 high-risk class. The morbidity and mortality rates for each EmSFI class observed in the development group are reported in Table 5. The overall mortality rate was similar to what recorded in the EmSFI 4–7 class. The difference did not reach statistical significance (odds ratio 0.664 [95% CI 0.450–0.984]). The overall morbidity rate was slightly lower than in the moderate risk class (*p* < 0.02; OR 1.386 [95% CI 1.058–1.815]). When excluding the minor complications, the overall morbidity rate was similar to what observed in the EmSFI 4–7 moderate risk class.

**Table 5:** Mortality and morbidity rate in development set

EmSFI risk class	Mortality Dv group *n*. (%)	Morbidity Dv group *n*. (%)	Clavien II–IV Dv group *n*. (%)
EmSFI 1–3	17/416 (4.1%)	105/416 (25.2%)	80/416 (19.2%)
EmSFI 4–7	45/312 (14.4%)	128/312 (41.0%)	87/312 (27.9%)
EmSFI 8–14	17/56 (30.4%)	29/56 (51.8%)	23/56 (41.1%)
Total	79/784 (10.1%)	262/784 (33.4%)	190/784 (24.2%)

### Validation cohort results

**Fig. 4:**
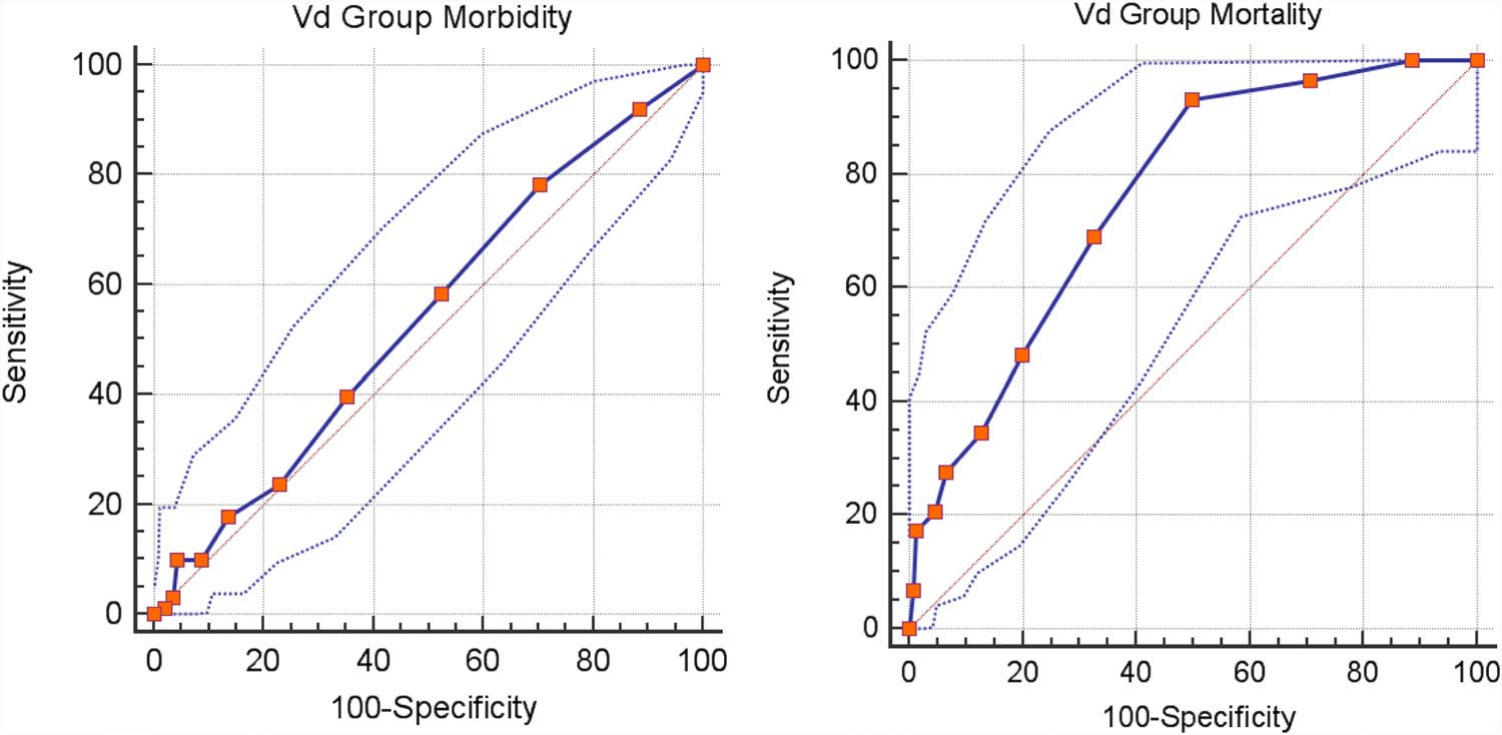
EmSFI ROC Curve of Morbidity (left) and Mortality (right) in Vd set

The model exhibited good discrimination ability (AUC = 0.762 [95% CI 0,682–0,842]) (Fig. 4) and good calibration (Hosmer–Lemeshow chi-square = 7,238; *p* = 0.299), reflecting a very good agreement between prediction by the final model and actual observation as regard to mortality in the validation cohort.

As concern morbidity the model showed a fair discrimination ability (AUC = 0.541 [95% CI 0.593–0.673]) (Fig. 4) and good calibration (Hosmer–Lemeshow chi-square = 4.022; *p* = 0.674), reflecting a poor agreement between prediction by the final model and actual observation.

The morbidity and mortality rates for each EmSFI class observed in the validation cohort are reported in Table 6. As seen in the development cohort, the overall mortality rate was similar to what recorded in the EmSFI 4–7 class (*p* = ns; OR 0.665 [95% CI 0.450–0.984]). With regard to morbidity, the EmSFI did not discriminate different risk classes except when limited to Clavien–Dindo III–IV complications.

**Table 6:** Mortality and morbidity rate in validation set

EmSFI risk class	Mortality Vd group *n*. (%)	Morbidity Vd group *n*. (%)	Clavien II–IV Vd group *n*. (%)	Clavien III–IV Vd group *n*. (%)
EmSFI 1–3	2/108 (1.9%)	42/108 (38.9%)	34/108 (31.5%)	20/108 (18.5%)
EmSFI 4–7	19/110 (17.3%)	49/110 (44.5%)	35/110 (31.8%)	28/110 (25.6%)
EmSFI 8–14	8/22 (36.4%)	10/22 (45.5%)	8/22 (36.4%)	8/22 (36.4%)
Total	29/240 (12.1%)	101/240 (42.1%)	77/240 (32.1%)	58/240 (24.1%)

## Discussion

Over the last decades, frailty has gradually acquired a well-defined clinical significance, evident right from the emerging of several operational definitions since the frailty phenotype proposed by Fried [15]. Despite the blossoming of different assessment tools, none of these gained a univocal consensus and no gold standard measure of frailty exists, showing the wide clinical range in which frailty syndrome occurs.

Unlike the spectrum of applicability of these tools, an agreement was established upon frailty definition: it is a clinical syndrome characterized by an increased vulnerability to stressors, associated with functional impairment and adverse outcomes [20].

The geriatric field has represented the main area of investigation on such status [21], but different specialties have adapted frailty assessment into their clinical practice. With regard to surgery, various scoring systems were employed, demonstrating a strong association between a frail profile and increased length of stay, postoperative complications, mortality rates and discharge to rehabilitation facilities [5–7].

Concerning specifically to general surgery, it has been demonstrated that in the elective setting a careful multidisciplinary preoperative assessment of elderly patients improves postoperative outcomes, but it is undoubtedly worthy to perform in case of older patients undergoing urgent procedures [22–24]. It is obvious that the decision-making process in such instances should be preceded by an accurate and less time-consuming assessment, even more so if we are faced with a vulnerable patient weakened by comorbidities [25]. The evaluation of surgical risks plays a pivotal role and the reliability of the so-called “gut-feeling” has not been no longer suitable due to its lack of reproducibility and, thereby, it has been upgraded with a lot of risk stratification tools developed across the years and validated in different medical and surgical populations [26, 27]. Nevertheless, there is no an ideal model particularly when elderly frail patients were considered. Most of the previously emerged prediction tools cannot be easily employed in the preoperative assessment. Moreover, their accuracy showed variability in relation to surgical specialty in which it has been tested. But more importantly, many of these models are still cumbersome and include a lot of variables in their scoring algorithms proving to be difficult to use at the bedside, time-consuming and face restrictions when incorporated into surgical evaluation and management [28–34]. This topic has been recently well addressed by Barbagallo which advocated the need to use simple tools for the evaluation of frailty and vulnerability in the surgical risk assessment [35].

It is with this awareness that we have decided to get involved in the FRAILESEL (Frailty and Emergency Surgery in the Elderly) multicenter study, with the aim of analyzing the most heterogeneous sample as possible of older surgical patients, developing and validating a simple, accurate and feasible risk assessment tool, which could be used to evaluate highly vulnerable individuals in case of both emergent and elective surgery. As a matter, the first variable of paramount importance in our analysis is represented by the surgical setting. It is known that emergency surgery in elderly patients has resulted in prolonged length of stay [36] and higher mortality and morbidity rate compared with elective procedures. Indeed, literature data have shown mortality rate ranging from 14 to 31% in the subset of elderly patients undergoing emergency procedures [37–40].

Among other selected predictive factors, age surely represents one of the most common parameters that affect morbidity and mortality. It is certainly true that the prevalence of frailty increases with age, as it is seen in 26% of patients aged 80 years or older compared with 7% of adults aged between 65 and 75 years [41, 42]. According to the related literature, we highlighted that octogenarians enrolled in our study have demonstrated a higher vulnerability with an increasing risk of morbidity and mortality onset. However, recent findings have assessed that older patients with the same chronological age could have divergent outcomes and, therefore, an objective measure of patient’s functional reserves becomes fundamental in predicting postsurgical morbidity and mortality rates [38].

Another issue to be considered is the association between the inflammatory status and frailty. Thus, we have extrapolated easily available data from patients’ charts, such as temperature, heart rate, respiratory rate and white blood cells count, reflecting SIRS hematochimic and clinical profile (that has been detected in almost 20% of patients in our series). Concerning this matter, last emerging evidences have suggested that the immune system undergoes several alterations in frail individuals, leading to a chronic inflammatory condition (“InflammAging”). The immune dysregulation characterized by higher levels of pro-inflammatory cytokines along with malnutrition are associated with different age-related pathophysiologic processes affecting outcomes such as sarcopenia, atherosclerosis, osteoporosis, functional decline, and disability [43, 44].

Moreover, investigating the impact of comorbidities on clinical outcomes of older surgical patients, we clearly defined the role of both cardiovascular and respiratory disease in affecting perioperative risk, often limiting the indications for surgical treatment. On the other hand, several chronic comorbid conditions (such as diabetes) that might increase the risk of postoperative complications and mortality [45], did not show a predictive role as single variable in this model and were consequently combined in a more significant item named as “Other comorbidities”.

Similar to what developed by Subramanian et al. [46], with the so-called five factors modified Frailty index, and by Revenig et al. [47], we provided a fast and time-sparing scoring system, also retracing some of the Fried’s criteria. Hence, mobility alterations and functional impairments were identified using clinical examination along with anamnestic data and/or information supplied by caregivers, avoiding measurements that require certain time to be performed.

We obtained a statistically based weight measure of preoperative variables significantly associated with the risk of adverse outcomes. The developed tool has been called EmSFI which is the acronym of Emergency Surgery Frailty Index. Despite the probably confounding label, our EmSFI is not actually a mere measure of frailty, but it is rather an elderly risk score based on possible frail profile and global deficit accumulation.

Guided by linear correlation and logistic regression analysis, we were able to stratify our study population in three risk groups according to the EmSFI value, observing a linear relationship between mortality rates and groups, among which the EmSFI 4–8 moderate risk class has shown mortality rate comparable to that registered among the entire study sample, consistently with literature data.

When the heterogeneous development set was fully investigated, the EmSFI score disclosed viability as predictor of mortality, whereas it lost its accuracy concerning to morbidity, showing one of the most important current limitations of this study maybe due to the wide spectrum of surgical procedures collected in our study. In fact, as Strasberg and colleagues well explained [48], it is highly difficult to obtain a precise morbidity grading irrespectively of the type of procedure performed, particularly regarding moderate to severe complications.

Precisely to this end, it would be necessary to adopt a more comprehensive system to ameliorate the pondering of morbidity burden, for instance, on the traces of the grading system developed by the U.S. National Institutes of Health (NIH) with the Common Terminology Criteria for Adverse Events (CTCAE) [49].

These theories then found their mainstay right on the analysis of the validation set. In fact, we validated our model on a subset of older patients that underwent surgical procedures for both benign and malignant colonic diseases. Colonic surgery is performed in 10–25% of abdominal emergencies in geriatric patients and is burdened by postoperative complications occurring in about one-third of patients undergoing colorectal resections [50, 51].

Also in the validation cohort, our EmSFI score showed a good accuracy in predicting mortality whereas it turned out to be not significant in determining the risk of developing postoperative complications. Indeed, we observed a slight gain of predictability within morbidity if just Clavien III–IV grades were considered.

### Limitations of the study

As clearly stated above, there are some limitations in this study. The first evidence is the difficulty to assess frailty in emergency setting by using the Fried’s criteria in accordance to the current international guidelines. The second one consists in a low accuracy of EmSFI as predictor of postoperative morbidity and in the necessity for an external validation in order to improve this prediction model. There are several factors potentially influencing postoperative morbidity. Among these, it needs to consider the cognitive status and the mobility that could affect the patient’s autonomy in the postoperative course. The evaluation of cognitive impairment based on reported data by relatives and/or caregivers and the lack of an objective measurement of walking speed might underestimate the real incidence of such variables altering their true statistical weight. In addition, mortality and morbidity may depend not only on patient’s intrinsic factors, but also on extrinsic determinants such as the time to surgery, the duration of procedure, and intraoperative findings. In our series, time to surgery and intraoperative blood loss were factors not statistically associated with morbidity, while the duration of surgery was not considered because not always recorded.

Furthermore, different diseases in the same organ as well as the same procedure with different surgical approach are burdened by different morbidity and mortality rates [12].

However, the multicenter design of this study with a well pre-defined protocol and homogeneous data collection method definitely provided a valid population for analysis.

Nonetheless, further investigations testing larger samples of patients are needed to deploy this tool according to various emergency surgery scenarios by considering also the duration of surgery. Additional efforts are also required to investigate specific surgical procedures and organ diseases to rank other items able to improve the accuracy in predicting complications that might be procedure or organ related.

Moreover, because we considered that EmSFI risk score could be applicable even in the context of elective surgery supplementary studies are needed to validate this tool also in such surgical setting.

## Conclusions

Frailty assessment has become pivotal in predicting risk of post-operative complications among elderly patients and it is more significant in emergency surgical settings.

Our results contribute to provide an effective surgical risk stratification and EmSFI represents a valid simple instrument to perform preoperative evaluations with moderate accuracy, improving perioperative risk management in elderly patients. The burden of studies such as this corroborates the importance of defining and measuring frailty status in older surgical patients to perform a tailored approach to patient’s treatment, considering alternative low-risk surgical or non-surgical options, and to provide an appropriate informed consent with an accurate individualized risk estimation for postoperative outcomes.
